# Association of Methylenetetrahydrofolate Reductase Gene Polymorphism in Mothers With Adverse Clinical Outcomes in Neonates

**DOI:** 10.7759/cureus.38001

**Published:** 2023-04-23

**Authors:** Divya D Panigrahi, Suprava Patel, Sarita Rajbhar, Phalguni Padhi, Seema Shah, Rachita Nanda, Eli Mohapatra

**Affiliations:** 1 Medicine, All India Institute of Medical Sciences, Raipur, IND; 2 Biochemistry, All India Institute of Medical Sciences, Raipur, IND; 3 Obstetrics and Gynaecology, All India Institute of Medical Sciences, Raipur, IND; 4 Neonatology, All India Institute of Medical Sciences, Raipur, IND

**Keywords:** methylenetetrahydrofolate reductase, preterm delivery, lbw, iugr, neonatal sepsis, neonatal death, congenital anomalies, c677t and a1298c, maternal mthfr

## Abstract

Background: The presence of polymorphic methylenetetrahydrofolate reductase (MTHFR) in mothers poses a risk for numerous detrimental outcomes in neonates. The present study investigated the association of maternal MTHFR A1298C and C677T single nucleotide polymorphisms (SNPs) with the clinical outcomes in their neonates.

Materials and methods: The cross-sectional study included 60 mothers and their neonates. Blood samples from mothers were analyzed for MTHFR A1298C and C677T SNP genotyping by real-time polymerase chain reaction. Clinical details of mothers and neonates were documented. Study groups were stratified based on wild, heterozygous, and mutant genotypes for the respective polymorphisms observed in mothers. Multinomial regression was applied for the association, followed by gene model formulation to estimate the impact of the genetic variants on the outcomes.

Results: The frequency percentages of mutant CC1298 and TT677 genotypes were 25% and 8.06%, respectively, and the mutant allele frequencies (MAF) were 42.5% and 22.5%. Percentages of adverse outcomes such as intrauterine growth restriction, sepsis, anomalies, and mortality were higher in neonates born to mothers with homozygous mutant genotypes. Maternal C677T MTHFR SNPs revealed a significant association with neonatal anomalies (p = 0.001). The multiplicative risk model depicted OR (95% CI) for CT vs. CC+TT as 3.0 (95% CI: 0.66-13.7), and for TT vs. CT+CC was 15 (95% CI: 2.01-112.12). The C677T SNP in mothers predicted a dominant model for neonatal death (OR (95% CI): 5.84 (0.57-60.03), p = 0.15), whereas the A1298C reported recessive model for 1298CC mothers (OR (95% CI): 11 (1.05-115.5), p = 0.02). Both the genotypes assumed a recessive model for adverse neonatal outcomes: OR (95%CI) for CC vs. AA+AC was 3.2 (0.79-12.9, p = 0.1), and for TT vs. CC+CT was 5.48 (0.57-175.7, p = 0.2). The risk for sepsis in neonates was nearly six times higher in those born from mothers with homozygous CC1298 and TT677 than in the wild and heterozygous variants.

Conclusion: Mothers with C677T and A1298C SNPs are highly susceptible to adverse outcomes in their neonates. Hence, screening the SNPs during the antenatal period can purposefully serve as a better predictive marker, following which proper clinical management could be planned.

## Introduction

The enzyme 5,10-methylenetetrahydrofolate reductase (5,10-MTHFR) catalyzes the reduction of 5,10-methylene tetrahydrofolate to 5-methyltetrahydrofolate (5-MTHF). The essential role of 5,10-MTHFR in the homocysteine-methionine cycle and S-adenosyl methionine (SAM) formation is well known. SAM is a donor of a methyl group vital for various metabolically demanding trans-methylation reactions, including the methylation of deoxyribonucleic acid (DNA) [1]. These reactions are essential for modulating the functionalities of protein and nucleic acid involved in regulating gene expressions like DNA hyper- and hypo-methylation, which have been extensively studied for various gene expressions and genome imprinting. Therefore, reduced activity of the enzyme creates a state of folate deficiency, which is crucial as it results in altered gene expression consequent to impaired DNA methylation and oxidative stress owing to homocysteinemia [2].

Polymorphism in the methylenetetrahydrofolate reductase (MTHFR) gene has been under much research in the last few decades. The common single nucleotide polymorphisms (SNPs) observed are C677T (cytosine by thymine at position 677 of MTHFR gene, valine replaces alanine at position 222 of MTHFR protein) and A1298C (adenine by cytosine at position 1298 of MTHFR gene, glutamic acid to alanine at position 429 of MTHFR protein) [3]. The frequency of these variants in the Indian population is not uncommon and is reported to vary from nearly 2% to 24% for 677T and 19% to 44% for 298C [1,4]. The variant form of the enzymes demonstrates reduced activity [5]. The enzyme, a key determinant for one-carbon transfer reactions involving active folate and vitamin B12, is vital for DNA synthesis and repair mechanisms, especially during implantation, fetal organogenesis, and in-utero development. Hence, women taking folic acid supplements are said to be protected from neural tube defects (NTDs). Yet, few mothers fail to be benefitted from folic acid supplementation and have offspring with NTDs [6,7]. In addition, these variants have been implicated in raised plasma homocysteine, leading to endothelial damage resulting in thromboembolic risk [8]. MTHFR polymorphisms and altered homocysteine metabolism are thus considered potential risk factors for impaired fetal perfusion. This pathophysiology in pregnant women often leads to obstruction in placental blood vessels, recurrent abortions, intrauterine growth restriction (IUGR), and fetal anomalies [8-10]. Studies have reported a significant association of MTHFR C677T and A1298C variants with NTDs, congenital heart disease (CHD), congenital anomalies such as Down syndrome, preterm birth, low birth weight (LBW), IUGR, and various other adverse birth outcomes [3,7,11-13]. Therefore, the candidate genes involved in these metabolic pathways should be explored to identify the associated genetic risks. It is speculated that MTHFR polymorphism might be a potential genetic risk for adverse neonatal outcomes but with inconsistent findings [3]. Therefore, the present study aimed to investigate the association of MTHFR gene variants, A1298C and C677T, in pregnant women with adverse outcomes of their neonates.

## Materials and methods

The cross-sectional study involved 60 adult women and their neonates. Mothers and their neonates admitted within one month of delivery were included in the study. Mothers with any history of smoking, high body mass index, gestational diabetes, preeclampsia, hypertension, sickle cell disease, other hemoglobinopathies, any other acute or chronic diseases or infections such as toxoplasmosis, rubella, cytomegalovirus, herpes simples (TORCH), tuberculosis, human immunodeficiency virus (HIV), human papillomavirus (HPV), or any other infection at any time of antenatal period were excluded from the study. The Institute Ethics Committee approved the study, and the participants were enrolled following written informed consent. Blood samples from mothers only were collected in the ethylenediaminetetraacetic acid (EDTA) vial. As per the case record form, all clinical details of the mother and the neonate were entered. The DNA extraction and Taqman-based SNP genotype assay by polymerase chain reaction (PCR) were processed per the manufacturer’s instructions using the MTHFR Genotyping Kit from Mylab Solutions, Pune, India [14]. The study group was stratified based on wild, heterozygous, and mutant genotypes for the respective polymorphisms observed in the maternal population. The wild, heterozygous, and mutant genotypes for A1298C were AA1298, AC1298, and CC1298, respectively. For C677T, the respective genotypes considered were CC677, CT677, and TT677, respectively. A1298 and C677 alleles are denoted as the wild (major) alleles. Similarly, the respective mutant (minor) alleles were C1298 and T677. The different genotypic categorization of the MTHFR SNPs used in this study is delineated in Table 1.

**Table 1:** The different genotypic categorizations of MTHFR SNPs (A1298C and C677T) in the present study MTHFR: methylenetetrahydrofolate reductase; SNPs: single nucleotide polymorphisms.

	MTHFR SNPs	Genotypes			Alleles	
		Mutant	Heterozygous	Wild	Mutant (minor)	Wild (major)
1	A1298C	CC1298	AC1298	AA1298	C1298	A1298
2	C677T	TT677	CT677	CC677	T677	C677

Statistical analysis

We performed the statistical analysis in IBM SPSS version 20 (IBM Corp., Armonk, NY). The frequency percentage distribution of genotypes was computed in the study population. For percentage calculation for genotype frequency, the total study population considered was 60; for allelic frequency, the total allele population considered was 120 (60*2). Multinomial logistic regression was performed to assess the association of the MTHFR C677T and A1298C genotypes of the mothers with the clinical outcomes of their neonates and interpreted with an odds ratio (OR) with a 95% confidence interval (95% CI). The variables showing OR more than two were further analyzed for gene models. The neonates without any altered outcome were considered normal, and accordingly, the gene model strategy was applied to estimate the impact of the homozygous, heterozygous, and mutant genotypes of A1298C and C677T of mothers on the outcome variables of their neonates [15]. The risk of the wild (major) and mutant (minor) alleles of mothers on the outcome variables of the neonates was evaluated by binary logistic regression. The statistical significance was considered for p < 0.05.

## Results

**Figure 1:**
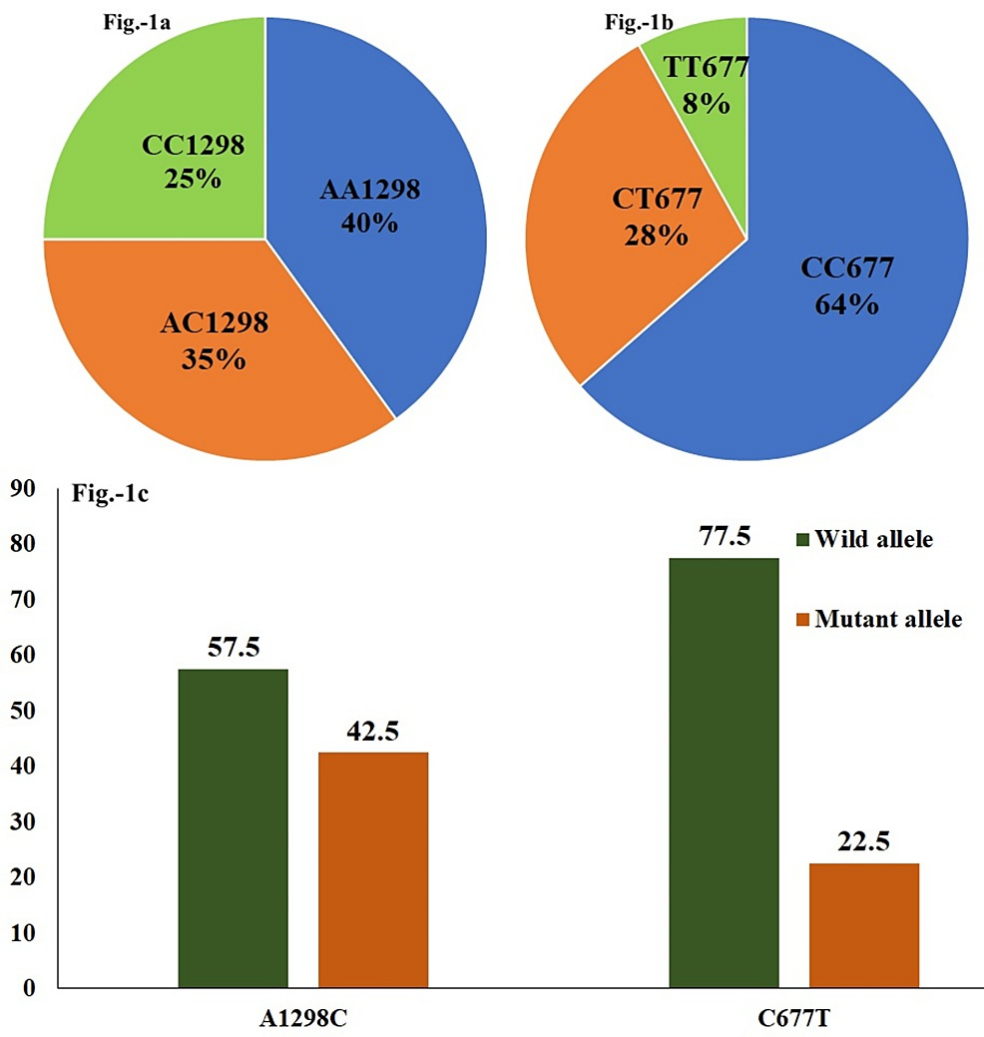
Percentage distribution of the MTHFR A1298C and C677T genotypes and the alleles in enrolled mothers Image A denotes A1298C genotypes, image B denotes C677T genotypes, and image C denotes wild and mutant alleles of both SNPs. MTHFR: methylenetetrahydrofolate reductase; SNPs: single nucleotide polymorphisms.

The frequency percentages of wild, heterozygote, and mutant forms for A1298C MTHFR polymorphism in the study population were, respectively, 40% (n = 24), 35% (n = 21), and 25% (n = 15) (Figure 1A). The respective frequency percentages for C677T MTHFR genotypes were 63.3% (n = 38), 28.3% (n = 17), and 8.06% (n = 5) (Figure 1B). The mutant allele frequencies (MAFs) were 42.5% (n = 51) and 22.5% (n = 27) for C1298 and T677, respectively (Figure 1C).

**Figure 2:**
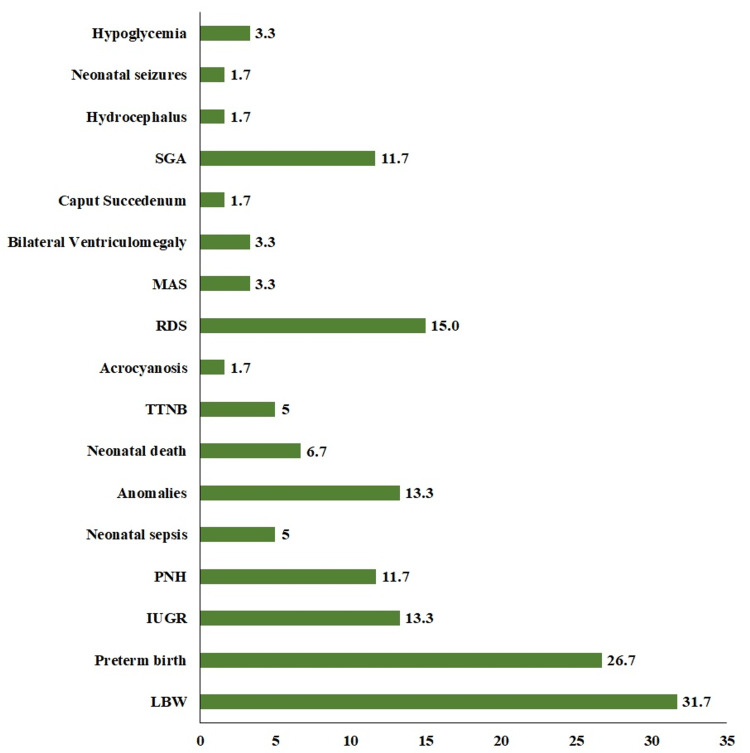
Frequency percentages of clinical presentations of the neonates included in the study (N = 60) Anomalies included congenital heart disorder (n = 2), Downs’ syndrome (n = 1), cleft palate (n = 2), lumbar meningomyelocele (n = 1), spina bifida (n = 1), and agenesis of corpus callosum (n = 1). LBW: low birth weight; IUGR: intrauterine growth restriction; PNH: prolonged neonatal hyperbilirubinemia; TTNB: transient tachypnea of the newborn; RDS: respiratory distress syndrome; MAS: meconium aspiration syndrome; SGA: small for gestational age.

In the neonatal population, the overall prevalence of LBW was 31.7%, preterm born were 26.7%, IUGR was diagnosed in 13.3%, congenital/chromosomal anomalies were observed in 13.3%, prolonged hyperbilirubinemia in 11.7%, neonatal death seen in 6.7%, and neonatal sepsis in 5% (Figure 2).

The distribution of maternal MTHFR variants and their association with the outcome variables in neonates is deciphered in Table 2. It was noted that neonates of 80% of mothers with CC1298 and 100% with TT677 genotypes were diagnosed with one or the other complications enlisted in Figure 2 (A1298C: p = 0.24, C677T: p = 0.18) (Table 2). Maternal allelic frequency distribution and their association with the outcome variables in neonates are deciphered in Table 3. The presence of either of the mutant alleles in mothers increases the risk of neonatal complications by nearly two times: A1298C - OR (95% CI): 1.96 (0.91-4.22); C677T - OR (95% CI): 2.06 (0.79-5.36) (Table 3).

**Table 2:** Distribution of maternal MTHFR SNPs and their association with the outcome of the neonates (N = 60) "N" denotes the study population, "n" denotes the number of neonates with or without the outcome variable, and "n (%)" denotes column percentage. P < 0.05 is considered significant. CC1298 denotes the variant form, AC1298 denotes the heterozygous form, and AA1298 denotes the wild form of A1298C MTHFR SNP. TT677 denotes the variant form, CT677 denotes the heterozygous form, and CC677 denotes the wild form of C677T MTHFR SNP. MTHFR: methylenetetrahydrofolate reductase; SNP: single nucleotide polymorphism.

MTHFR SNPs	A1298C genotypes					C677T genotypes			
	CC1298 (n = 15)	AC1298 (n = 21)	AA1298 (n = 24)	Chi-square (p-value)	TT677 (n = 5)		CT677 (n = 17)	CC677 (n = 38)	Chi-square (p-value)
Neonatal outcomes	n (%)	n (%)	n (%)		n (%)		n( %)	n (%)	
Neonatal complications (n = 37)	12 (80)	12 (57.1)	13 (54.2)	2.89 (0.24)	5 (100)		10 (58.8)	22 (57.9)	3.39 (0.18)
Apparently normal (n = 23)	3 (20)	9 (42.9)	11 (45.8)		0		7 (41.2)	16 (42.1)	

**Table 3:** Distribution of maternal MTHFR alleles and their association with the outcome of the neonates (N = 120) "N" denotes the total allelic population (2 x 60), "n" denotes the number of neonates with or without the outcome variable, and "n (%)" denotes column percentage. P < 0.05 is considered significant. C1298 denotes the variant allele and A1298 denotes the wild allele of A1298C MTHFR SNP. T677 denotes the variant allele and C677 denotes the wild allele of C677T MTHFR SNP. MTHFR: methylenetetrahydrofolate reductase; SNP: single nucleotide polymorphism.

MTHFR alleles	A1298C alleles			C677T alleles		
	C1298 allele (n = 51)	A1298 allele (n = 69)	Chi-square (p-value)	T677 allele (n = 27)	C677 allele (n = 93)	Chi-square (p-value)
Neonatal outcomes	n (%)	n (%)		n (%)	n (%)	
Neonatal complications (n = 74)	36 (70.6)	38 (55.1)	2.99 (0.08)	20 (74.1)	54 (58.1)	2.27 (0.13)
Apparently normal (n = 46)	15 (29.4)	31 (44.9)		7 (25.9)	39 (41.9)	
OR (95% CI)	1.96 (0.91-4.22)			2.06 (0.79-5.36)		

**Figure 3:**
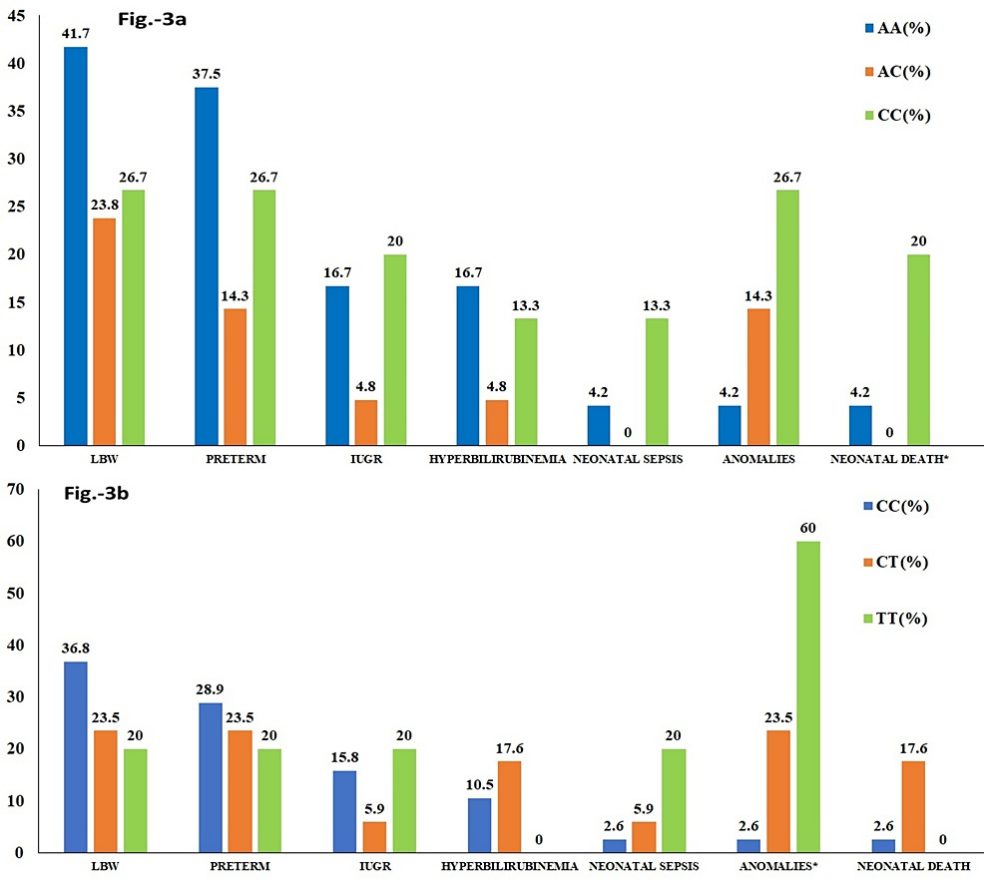
Frequency percentages of maternal MTHFR A1298C and C677T genotypes for the outcomes variables in their neonates (N = 60) Image A illustrates mothers with MTHFR A1298C genotypes. Image B illustrates mothers with MTHFR C677T genotypes. * denotes statistical significance at p < 0.05. MTHFR: methylenetetrahydrofolate reductase; LBW: low birth weight; IUGR: intrauterine growth restriction.

As delineated in Figure 3 and Table 4, it was observed that a higher percentage of mothers with mutant genotypes documented altered outcomes in their neonates, such as IUGR, neonatal sepsis, and congenital/chromosomal anomalies. Mothers with the CC1298 genotype reported a significant association with neonatal death (p = 0.049). Neonatal death was reported in 20% of mothers with homozygous mutant genotype as compared to only 4.2% with wild genotype (Figure 3A). Unlike A1298C, the maternal C677T MTHFR genotype revealed a significant association with congenital/chromosomal anomalies in neonates (p = 0.001). A total of 60% of TT677 and 23.5% of heterozygous CT677 had babies delivered with anomalies (Figure 3B). Similarly, neonates of 26.7% of mothers with CC1298 and 14.3% of mothers with AC1298 were diagnosed with anomalies (p = 0.13; Figure 3A). Neonates born to 13.3% and 20% of mothers with homozygous mutant genotypes developed sepsis within a month of delivery as against those born to 4.2% and 2.6% of mothers with wild genotypes (p = 0.19 for A1298C and p = 0.24 for C677T, respectively).

**Table 4:** Frequency distribution of maternal MTHFR genotypes and their association with various clinical outcome variables in neonates (N = 60) "N" denotes the study population, "n" denotes the number of neonates with or without the outcome variable, and "n (%)" denotes column percentage. * p < 0.05 is considered significant. CC1298 denotes the variant form, AC1298 denotes the heterozygous form, and AA1298 denotes the wild form of A1298C MTHFR SNP. TT677 denotes the variant form, CT677 denotes the heterozygous form, and CC677 denotes the wild form of C677T MTHFR SNP. MTHFR: methylenetetrahydrofolate reductase; SNP: single nucleotide polymorphism; IUGR: intrauterine growth restriction.

MTHFR SNPs		A1298C genotypes			Chi-square (p-value)	C677T genotypes			Chi-square (p-value)
		CC1298 (n = 15)	AC1298 (n = 21)	AA1298 (n = 24)		TT677 (n = 5)	CT677 (n = 17)	CC677 (n = 38)	
Clinical outcome variables		n (%)	n (%)	n (%)		n (%)	n (%)	n (%)	
Birth weight	Low (n = 19)	4 (26.7)	5 (23.8)	10 (41.7)	1.88 (0.39)	1 (20)	4 (23.5)	14 (36.8)	1.3 (0.52)
	Normal (n = 41)	11 (73.3)	16 (76.2)	14 (58.3)		4 (80)	13 (76.5)	24 (63.2)	
Preterm/term born	Preterm (n = 16)	4 (26.7)	3 (14.3)	9 (37.5)	3.09 (0.21)	1 (20)	4 (23.5)	11 (28.9)	0.3 (0.86)
	Term (n = 44)	11 (73.3)	18 (85.7)	15 (62.5)		4 (80)	13 (76.5)	27 (71.1)	
IUGR	Yes (n = 8)	3 (20)	1 (4.8)	4 (16.7)	2.14 (0.34)	1 (20)	1 (20)	6 (15.8)	1.21 (0.55)
	No (n = 52)	12 (80)	20 (95.2)	20 (83.3)		4 (80)	16 (94.1)	32 (84.2)	
Prolonged hyperbilirubinemia	Yes (n = 7)	2 (13.3)	1 (4.8)	4 (16.7)	1.59 (0.45)	0	3 (17.6)	4 (10.5)	1.29 (0.52)
	No (n = 53)	13 (86.7)	20 (95.2)	20 (83.3)		5 (100)	14 (82.4)	34 (89.5)	
Neonatal sepsis	Yes (n = 3)	2 (13.3)	0	1 (4.2)	3.33 (0.19)	1 (20)	1 (5.9)	1 (2.6)	2.85 (0.24)
	No (n = 57)	13 (86.7)	21 (100)	23 (95.8)		4 (80)	16 (94.1)	37 (97.4)	
Congenital/chromosomal anomalies	Yes (n = 8)	4 (26.7)	3 (14.3)	1 (4.2)	4.07 (0.13)	3 (60)	4 (23.5)	1 (2.6)	14.72* (0.001)
	No (n = 52)	11 (73.3)	18 (85.7)	23 (95.8)		2 (40)	13 (76.5)	37 (97.4)	
Neonatal death	Yes (n = 4)	3 (20)	0	1 (4.2)	6.03* (0.049)	0	3 (17.6)	1 (2.6)	4.65 (0.09)
	No (n = 56)	12 (80)	21 (100)	23 (95.8)		5 (100)	14 (82.4)	37 (97.4)	

**Figure 4:**
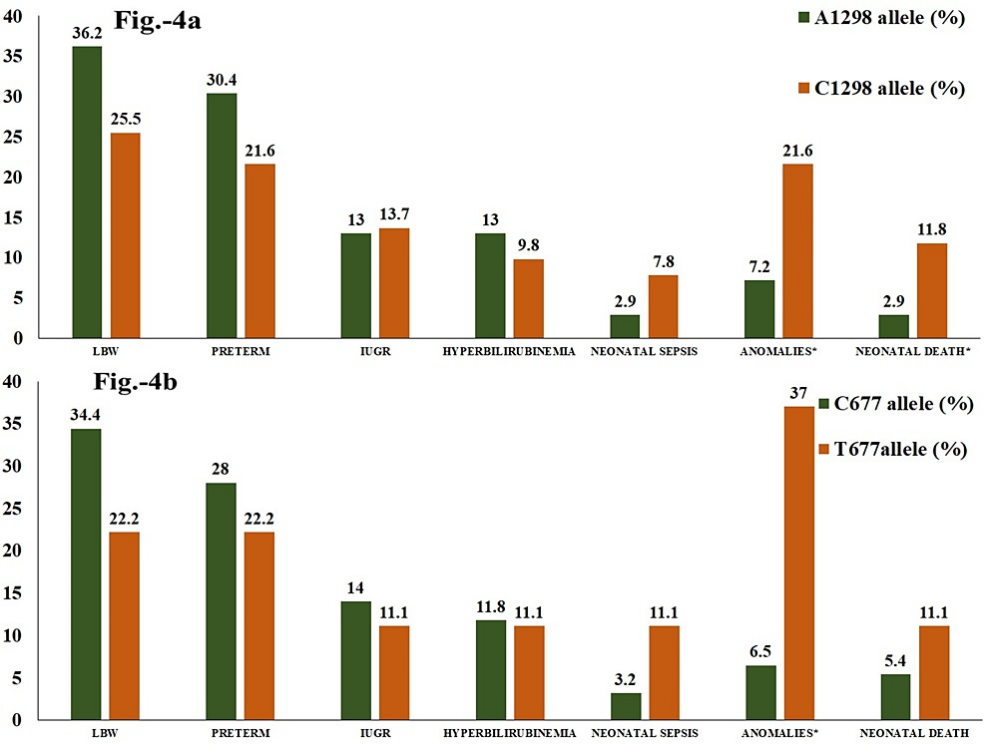
Frequency percentages of maternal MTHFR A1298C and C677T wild and mutant alleles for the outcomes variables in their neonates (N = 120) Image A illustrates mothers with MTHFR A1298C wild and mutant alleles, and image B illustrates mothers with MTHFR C677T wild and mutant alleles. * denotes statistical significance at p < 0.05. MTHFR: methylenetetrahydrofolate reductase; LBW: low birth weight; IUGR: intrauterine growth restriction.

Figure 4 and Table 5 illustrate the frequency percentages of sepsis, congenital/chromosomal anomalies, and mortality were higher in neonates born to mothers with mutant allele than mothers with wild allele. The risk for sepsis in neonates was nearly six times higher in those born to mothers with homozygous CC1298 (95% CI: 0.57-80.74, p = 0.09) and TT677 (95% CI: 0.48-89.8, p = 0.11) than the wild and heterozygous variants. Maternal C1298 and T677 allelic distribution were significantly associated with congenital/chromosomal anomalies in neonates (p = 0.023 and p < 0.001, respectively). The risk for diagnosing with anomalies was nearly 3.5 times (95% CI: 1.14-10.88) for C1298 allelic mothers and more than 8.5 times for T677 allelic mothers (95% CI: 2.73-26.61). Nearly 11% of the maternal mutant allelic population depicted neonatal death (p = 0.05 for C1298 and p = 0.29 for T677). The presence of mutant alleles in mothers raised the probability of neonatal mortality by 4.5 times for C1298 (95% CI: 0.86-23.1) and 2.2 times for T677 (95% CI: 0.49-9.87), as illustrated in Table 5.

**Table 5:** Frequency distribution of maternal MTHFR alleles and their association with various clinical outcome variables in neonates (N = 120) "N" denotes the total allelic population (2 x 60), "n" denotes the number of neonates with or without the outcome variable, and "n (%)" denotes column percentage. * p < 0.05 is considered significant. C1298 denotes the variant allele and A1298 denotes the wild allele of A1298C MTHFR SNP. T677 denotes the variant allele and C677 denotes the wild allele of C677T MTHFR SNP. MTHFR: methylenetetrahydrofolate reductase; SNP: single nucleotide polymorphism; IUGR: intrauterine growth restriction.

MTHFR alleles		A1298C alleles			C677T alleles		
		C1298 allele (n = 51)	A1298 allele (n = 69)	Chi-square (p-value)	T677 allele (n = 27)	C677 allele (n = 93)	Chi-square (p-value)
Clinical outcomes		n (%)	n (%)		n (%)	n (%)	
Birth weight	Low (n = 38)	13 (25.5)	25 (36.2)	1.56 (0.21)	6 (22.2)	32 (34.4)	1.44 (0.23)
	Normal (n = 82)	38 (74.5)	44 (63.8)		21 (77.8)	61 (65.6)	
	OR (95% CI)	0.602 (0.27-1.34)			0.55 (0.2-1.49)		
Preterm/term born	Preterm (n = 32)	11 (21.6)	21 (30.4)	1.18 (0.28)	6 (22.2)	26 (28)	0.35 (0.55)
	Term (n = 88)	40 (78.4)	48 (69.6)		21 (77.8)	67 (72)	
	OR (95% CI)	0.63 (0.27-1.46)			0.74 (0.27-2.03)		
IUGR	Yes (n = 16)	7 (13.7)	9 (13)	0.012 (0.91)	3 (11.1)	13 (14)	0.149 (0.7)
	No (n = 104)	44 (86.3)	60 (87)		24 (88.9)	80 (86)	
	OR (95% CI)	1.06 (0.37-3.07)			0.77 (0.2-2.93)		
Prolonged hyperbilirubinemia	Yes (n = 14)	5 (9.8)	9 (13)	0.299 (0.59)	3 (11.1)	11 (11.8)	0.01 (0.92)
	No (n = 106)	46 (90.2)	60 (87)		24 (88.9)	82 (88.2)	
	OR (95% CI)	0.73 (0.23-2.31)			0.93 (0.24-3.6)		
Neonatal sepsis	Yes (n = 6)	4 (7.8)	2 (2.9)	1.51 (0.22)	3 (11.1)	3 (11.1)	2.74 (0.098)
	No (n = 114)	47 (92.2)	67 (97.1)		24 (88.9)	90 (96.8)	
	OR (95% CI)	2.85 (0.5-16.21)			3.75 (0.71-19.77)		
Congenital/chromosomal anomalies	Yes (n = 16)	11 (21.6)	5 (7.2)	5.21* (0.023)	10 (37)	6 (6.5)	16.94* (p<0.001)
	No (n = 104)	40 (78.4)	64 (92.8)		17 (63)	87 (93.5)	
	OR (95% CI)	3.52 (1.14-10.88)			8.53 (2.73-26.61)		
Neonatal death	Yes (n = 8)	6 (11.8)	2 (2.9)	3.71* (0.05)	3 (11.1)	5 (5.4)	1.11 (0.29)
	No (n = 112)	45 (88.2)	67 (97.1)		24 (88.9)	88 (94.6)	
	OR (95% CI)	4.46 (0.86-23.1)			2.2 (0.49-9.87)		

**Figure 5:**
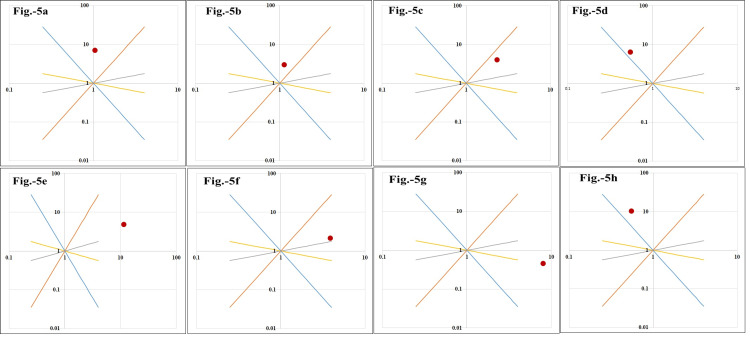
Genetic models depicting the risk of C677T and A1298C genotypes for the outcome variables in the neonates born to the mothers enrolled for the study (N = 60) Image A denotes the recessive model of C677T for neonatal complications; B denotes the recessive model of A1298C for neonatal complications; C denotes the additive model of C677T for neonatal sepsis; D denotes the recessive model of A1298C for neonatal sepsis; E denotes the multiplicative model of C677T for neonatal anomalies; F denotes the recessive model of A1298C for neonatal anomalies; G denotes the dominant model of C677T for neonatal death; H denotes the recessive model of A1298C for neonatal death.

The graphs for the gene model depicting the risk of maternal C677T and A1298C genotypes for the outcome variables in the neonates born to the mothers enrolled for the study are reflected in Figure 5. Both the genotypes assumed a recessive model for the presence of any of the neonatal complications (Figures 5A, 5B). OR (95% CI) for CC vs. AA+AC was 3.2 (0.79-12.9), and for TT vs. CC+CT, it was 5.48 (0.57-175.7). It signified that mothers with mutant genotypes (CC1298 or TT677) were three to five times more prone for their neonates to develop an altered outcome. For neonatal sepsis C677T genotypes, mothers revealed an additive model (OR (95% CI) CT+TT vs. CC = 3.7 (0.32-43.37)), whereas, for A1298C, the noted OR (95% CI) for CC vs. AC+AA (6.7 (0.57-80.74)) reflected recessive model for the risk (Figures 5C, 5D). The SNPs' models were the multiplicative type for congenital/chromosomal anomalies (Figures 5E, 5F). The odds for CT vs. CC+TT was 3.0 (95% CI: 0.66-13.7), and that for TT vs. CT+CC was 15 (95% CI: 2.01-112.12). Similarly, for A1298C variants, the OR (95% CI) for AC vs. AA+CC was 1.13 (0.24-5.29), and for CC vs. AA+AC, it was 3.73 (0.8-17.34). The C677T SNP in mothers predicted a dominant model for neonatal death (OR (95% CI): 5.84 (0.57-60.03)) in the presence of mutant allele, whereas the A1298C reported a recessive model so that neonates of homozygous 1298CC mothers were 11 times more prone for mortality (95% CI: 1.05-115.5) (Figures 5G, 5H).

The neonatal outcomes such as LBW, preterm birth, IUGR, and hyperbilirubinemia documented odds of less than one and thus implied that maternal mutant alleles have the most negligible influence on these variables. However, the genotypic distribution showed a very insignificant recessive model for IUGR for mothers homozygous for CC vs. AC+AC (OR (95% CI): 1.9 (0.42-9.6)) and TT vs. CC+CT (OR (95% CI: 1.64 (0.05-15.02)).

## Discussion

The enzyme MTHFR plays a central catalytic role in DNA synthesis and repair during the cell cycle and cellular differentiation during fetal development. MTHFR SNPs are not uncommon in our population. Hence, the study was undertaken to delineate the significant genetic susceptibility of altered outcomes in neonates. Mothers exhibiting polymorphism in the gene coding MTHFR, such as C677T and A1298C SNPs, make them highly susceptible to adverse neonatal outcomes. The SNPs significantly increased the risk for congenital/chromosomal anomalies, development of sepsis, and mortality risk in their neonates. The gene models predicted that neonates born to mothers homozygous for the mutant alleles were more for adverse outcomes.

The frequency percentages of the homozygous variant genotypes CC1298 and TT677 were 25% and 8.06%, respectively, corroborated with the previously reported frequency of 19.7% and 2%, respectively, by Patel et al.'s study in this area [4]. Angeline et al. recorded frequencies of 15.3% for CC1298 and 1.38% for TT677, and the MAF were 38.9% and 10.4%, respectively [16]. Similarly, the MAF in Kumar et al.'s study was 44% and 15% [17]. The respective MAFs recorded in the present study were 42.5% and 22.5%. The mutant allele percentage distribution among various regions in India varied nearly from 2% to 24% for T677 and 19% to 44% for C1298 [1,4]. The frequency distribution difference might reflect the distribution pattern in different geographical regions [17,18].

MTHFR is the critical enzyme for maintaining the active folate level and one-carbon transfer crucial for DNA methylation, synthesis, and repair. Reduced activity due to polymorphism minimizes the synthesis of 5-MTHF, essentially required for the remethylation of homocysteine to methionine and DNA methylation [7,11]. Polymorphism begets conformational changes in the binding site of the SAM that affects the methylation reactions. DNA methylation is crucial for regulating gene expression, which is imperative in implantation, apoptosis during organogenesis, and overall in-utero fetal development [6,11,12]. We observed that the presence of either mutant variants of A1298C and C677T in mothers recorded higher percentages of altered outcomes in their neonates. Of all the variables, anomalies such as NTD, CHD, patent ductus arteriosus (PDA), atrial septal defect (ASD), and Down syndrome were remarkably higher in neonates born to these mothers (Figures 3A, 3B). Recent studies demonstrated the association of MTHFR SNPs with congenital anomalies. Yan et al. stated MTHFR C677T is a genetic risk factor for NTDs. The meta-analysis depicted the risk to be twice more (2.022) in the presence of TT677 than CC677 (95% CI: 1.508-2.712) [7].

Similarly, Zhang et al. observed a significant association of C677T SNP with CHD. C677T variants depicted higher odds for both recessive (1.69) and dominant models (1.35) and homozygous and heterozygous models for CHD. However, A1298C MTHFR failed to significantly impact CHD except for the recessive model (OR = 1.42) [11]. Similarly, Yadav et al. reported that mothers with C677T polymorphism (OR (95% CI): 1.20 (1.13-1.28)) were potentially susceptible to giving birth to offspring with NTD, while mothers with A1298C polymorphism did not exhibit significant distribution [19]. Zhu et al. too corroborated a strong association of the TT677 genotype and T677 alleles with ASD and PDA [20]. The present study findings stand in agreement with the above for the SNPs. A strong association was evidenced for CC677 genotypic mothers with congenital/chromosomal anomalies in neonates such as CHD, meningomyelocele, spina bifida, cleft palate, and Down syndrome (p = 0.001, Table 4 and Figure 2).

Further, the study established a significant multiplicative risk for delivering babies with such anomalies if the mother has any one of the mutant alleles (Figures 5E, 5F). Like previous studies, the results also imply that MTHFR polymorphisms affect the methylase-specific enzymes, primarily involved in DNA synthesis and repair and eventually the fetal organogenesis and development in-utero. Thus, MTHFR C677T and A1298C SNPs play a potential role in decreased fetal viability and might be crucial for the in-utero survival of the fetus [11]. Folate and vitamin B12 are indispensable for genome stability. Animal model and cell culture studies have reflected DNA hypomethylation, chromosome breakage, and aneuploidy, as in Down syndrome and recurrent abortions, in folate depletion state [20,21]. At the same time, Saraswathy et al. observed higher odds for TT677 for recurrent miscarriages (OR (95% CI): 7.33 (0.48-111.2)), highlighting the role of hypermethylation of MTHFR C677T at specific promoter regions that might have a vital role in implantation or proper fetal development [10].

Hyperhomocysteinemia, the resultant metabolic effect of insufficient active folate, is also related to MTHFR mutations and congenital anomalies [8,22]. Homocysteinemia, in turn, increases oxidative stress and inflammatory cascade in the endothelial cells leading to vascular thrombosis, including placental thrombosis [9]. Thrombotic events during in-utero development lead to abnormal materno-fetal perfusion, eventually leading to intrauterine death (IUD), IUGR, LBW, or preterm delivery [3]. Conversely, few studies reported a lower risk or a protective influence for LBW or preterm delivery [23-25]. No association was observed for either maternal MTHFR SNPs for LBW or preterm deliveries (Figure 3 and Table 4). Instead, the data revealed a decreased risk of LBW and preterm delivery in the presence of mutant alleles T677 and C1298 (Figure 4 and Table 5), as reported by Nurk et al. and Resch et al. [24,26]. On the contrary, Tiwari et al.'s study comprising 209 cases of preterm deliveries concluded that the distribution of MTHFR mutant genotypes was higher in preterm cases and increased the risk of preterm delivery [27]. However, mothers with homozygous mutant genotypes for A1298C and C677T did show some risk (nearly 1.5 times) for IUGR in their neonates. The finding was equivocal to the report for elevated risk of IUGR in mothers with mutant T alleles (OR (95% CI: 1.2 (1.0-1.4); p = 0.04) by Nurk et al. [24]. This could be attributed to compromised materno-fetal circulation and placental vaso-occlusion following thrombosis as a result of reduced active folate and homocysteinemia. However, various studies attributed maternal MTHFR SNPs as potential genetic risk factors for adverse outcomes in neonates, but with inconsistent conclusions. The varied observations might be due to the differences in sample size, inclusion criteria, and study design applied for these studies. Further analysis estimated that the maternal mutant MTHFR forms magnified the risk of acquiring neonatal sepsis in their infants (Figure 5C, 5D), contrary to Zeeshan et al.'s study, which showed no risk [28]. Associated nutritional deficiency of folate and vitamin B12 in mothers due to polymorphic MTHFR, shall also be reflected in neonates [28]. These water-soluble vitamins are essentially required for adequate production and maturation of blood cells, including the immune cells [27,29]. The higher risk observed in this study for mortality in neonates born to mothers with mutant alleles could be multifactorial and majorly ascribed to the deficiency of micronutrients in neonates secondary to maternal MTHFR SNPs. We have not evaluated the nutritional status of mothers and neonates and thus, exclusive research needs to be conducted to establish the connecting link between mutant MTHFR genotypes and sepsis in infants. Studies have suggested that supplementation with L-methyl folate rather than conventional folic acid during the antenatal period would prevent the adverse outcome in fetuses and neonates born to mothers with MTHFR SNPs since methyl folate is the active form that becomes readily available to the mother and fetus to maintain the folic acid and homocysteine levels in mothers [30].

Limitation

The study's primary limitation was that it was a hospital-based cross-sectional study catering small sample size. Secondly, biochemical parameters like folic acid, vitamin B12, and homocysteine levels were not estimated, either in mothers or neonates, which could have added more insight regarding the biochemical changes associated with the genotypes and their impacts on neonates. Therefore, a well-designed study on large cohorts would enable a more accurate association of the genotypes with the outcome variables. Screening for MTHFR SNPs and biochemical parameters in the cohort of antenatal mothers at each trimester would provide more accurate analytical results regarding the association of these genotypes with fetal and neonatal health. Further, randomized clinical trials with active folate supplementation in one arm and conventional folic acid supplementation in the other arm provide more substantial evidence to derive an appropriate treatment protocol.

## Conclusions

The current study depicts the susceptibility of pregnant women with variant MTHFR A1298C and C677T genotypes to altered outcomes in neonates delivered, owing to a folate deficient state consequent to polymorphic MTHFR enzyme. Thus, the studied MTHFR SNPs considered a genetic risk factor for congenital/chromosomal anomalies, IUGR, sepsis, and mortality in their neonates, and antenatal screening for MTHFR SNPs could assess the high-risk mothers and initiate appropriate clinical interventions.

Highlights

The MTHFR A1298C and C677T gene polymorphisms in mothers could be ascribed as a genetic risk predictor for adverse outcomes in neonates. The present study expressed frequency percentages of 25% and 8.06% for mutant CC1298 and TT677 genotypes, respectively. Neonates born to mothers with mutant genotypes were more prone to complications like congenital/chromosomal anomalies, IUGR, sepsis, and mortality. Polymorphic MTHFR might result in a deficiency of active folate in mothers, eventually creating a lack of the micronutrient in their neonates. The mothers with the studied MTHFR SNPs might require dose modification of folic acid or active folate.
